# Web-Based Intervention for Postpartum Depression: Formative Research and Design of the MomMoodBooster Program

**DOI:** 10.2196/resprot.2329

**Published:** 2012-11-22

**Authors:** Brian G Danaher, Jeannette Milgrom, John R Seeley, Scott Stuart, Charlene Schembri, Milagra S Tyler, Jennifer Ericksen, Whitney Lester, Alan W Gemmill, Peter Lewinsohn

**Affiliations:** 1 Oregon Research InstituteEugene, ORUnited States; 2 Parent-Infant Research InstituteHeidelberg Repatriation HospitalVictoriaAustralia; 3 Department of PsychologyUniversity of MelbourneVictoriaAustralia; 4 Iowa Depression and Clinical Research CenterUniversity of Iowa Hospitals and ClinicsIowa City, IAUnited States

**Keywords:** postpartum depression, Web-based intervention, formative research

## Abstract

**Background:**

Postpartum depression is a significant public health problem affecting approximately 13% of women. There is strong evidence supporting Cognitive Behavioral Therapy (CBT) for successful psychosocial treatment. This treatment model combines cognitive and behavioral strategies to address pessimism, attributions for failure, low self-esteem, low engagement in pleasant activities, social withdrawal, anxiety, and low social support. Encouraging results have been reported for using Web-based CBT interventions for mental health domains, including the treatment of panic disorder, post-traumatic stress disorder, and complicated grief and depression. To date, however, Web-based interventions have not been used and evaluated specifically for the treatment of postpartum depression.

**Objective:**

We describe the formative work that contributed to the development of our Web-based intervention for helping to ameliorate symptoms of postpartum depression, and the design and key components of the program.

**Methods:**

A total of 17 focus group participants and 22 usability testers, who shared key characteristics with the participants of our planned feasibility study, took part.

The proposed structure and ingredients of the program and mock-ups of selected webpages were presented to focus group participants. At various points, participants were asked a series of thought questions designed to elicit opinions and set the occasion for group discussion. At the end of the session, participants were asked to describe their overall reaction to the proposed features of the program emphasizing candid opinions about what they did not like and features they thought were missing and should be added.

Usability testers were asked to interact with a series of seven different Web-based interactions planned for the program while receiving minimal direction. Each tester was asked to describe her thoughts using a *think-aloud* technique. They were then asked to consider all that they had learned about the program and complete the System Usability Scale that we adapted slightly to be appropriate for evaluating the proposed website.

Transcripts from the focus groups and usability tests were reviewed by research team members for overarching themes with particular emphasis on suggested changes. A list emerged, and iterative and incremental adjustments were made as a result.

**Results:**

The qualitative and quantitative data gathered in the focus groups and usability sessions reported here suggest that the new mothers involved had largely positive reactions to the major features of the program and that those program features performed well in terms of usability.

**Conclusions:**

An overview of the eventual design, architecture, and key program ingredients of the *MomMoodBooster* program is provided including innovative features supplementing 6 core CBT sessions, which include a partner’s website, a library, and individual feedback by a personal coach.

## Introduction

Postpartum depression (PPD) is a significant public health problem affecting many women with incidence estimates ranging from 5% to more than 13% of new mothers [1-3]. PPD causes significant suffering in women and their families and has an adverse impact on infant development [4-6].

Stuart, O’Hara, and Gorman [7] have classified two types of psychosocial interventions for postpartum mood disorders: (1) preventive programs introduced during pregnancy or early during the puerperium, and (2) interventions designed to help ameliorate the depressive symptoms experienced by women who have already developed postpartum depression (PPD). For a meta-analysis of psychosocial interventions for PPD, see Dennis and Hodnett [8]. Cognitive Behavioral Therapy (CBT) has been found to be a successful psychosocial treatment approach for depression [9-14].

Lewinsohn’s Coping With Depression (CWD) course [15,16] is a treatment model that combines cognitive and behavioral strategies to address pessimism, attributions for failure, low self-esteem, low engagement in pleasant activities, social withdrawal, anxiety, and low social support and has been adapted and evaluated in over 25 Randomized Controlled Trials (RCTs) in the treatment or prevention of depression [17]. Two meta-analyses [17,18] confirm the efficacy of the core CWD treatment approach and its adaptability for specific groups, reporting the average effect size (Cohen’s d) as ranging from .28 to .65. CBT has been widely disseminated [17,19], extensively evaluated with different populations (adolescents, adults, the elderly), and delivered via a range of modalities (individual, group, bibliotherapy, television, the Internet). Research results indicate that CBT is particularly helpful for individuals with mild to moderate depression [20,21].

Milgrom, Martin, and Negri [22] adapted Lewinsohn’s approach to create their Getting Ahead of Postnatal Depression program that included: (a) introducing behavioral activation (increasing pleasant activities) before presentation of cognitive strategies; (b) recommending relaxation “on the run” techniques; (c) reducing “homework”; (d) building support networks; and (e) incorporating partner sessions [22-25]. Milgrom’s program included three partner sessions due to the impact of postpartum depression on the relationship and the finding that poor dyadic adjustment is a risk factor [26,27]. The program also emphasizes the infant, due to the impact of postpartum depression on the infant and mother-infant relationship [28,29]. Content for the Partner Support Program and some library articles drew on other PIRI programs “Towards Parenthood” [25] and the Community HUGS Specialized Playgroup [30].

### Limited Utilization and Barriers to Treatment

It is becoming evident that even when postnatal depression is detected, use of clinic-based treatment for postnatal depression is poor, with only around 30-40% of women taking up treatment services [31-32]. Maternal beliefs and attitudes can form barriers that can significantly reduce the uptake of treatment for postpartum depression. One study [33] found that for many depressed new mothers, fear about acknowledging emotional distress (even to themselves) or admitting they may not be coping and the stigma associated with this leads them to “keep up appearances”. The end result is that they decide not to seek help. Practical aspects of help-seeking with a young baby (eg, travel, cost, tiredness, child care, organization, and lack of motivation) while suffering from the symptoms of depression further increase the barriers to treatment uptake [33,34]. Delivering CBT treatment via the Internet may well reduce these barriers and make helpful treatment more readily available and attractive. Moreover, Internet treatments enable mothers to reduce feelings of stigma by being somewhat anonymous, as they do not have to attend a clinic in person. Internet treatments can thus significantly extend the reach of treatment to mothers who may benefit but would not otherwise attend regular clinic treatment.

### Web-Based Approaches for Depression

Although early attempts to use Web-delivered interventions to reduce depressive symptomatology yielded equivocal results [35,36], subsequent studies have reported more promising findings [20,37-42]. It is important to note that encouraging results have also been reported for using Web-based CBT interventions for other mental health disorders including the treatment of panic disorder [43,44], post-traumatic stress disorder [45,46], and complicated grief [47]. To date, Web-based interventions have not been used and evaluated for the treatment of postpartum depression.

Web-based interventions offer unique advantages that should increase engagement and improve impact [48,49]:

- Web-based treatment programs can reach a larger percentage of women in need than clinic-based programs. This public health impact is especially important for women in rural settings with limited transportation, childcare resources, and access to mental health professionals [50,51].
- Program content can be tailored to participant characteristics and interests. While there are not yet clear data as to which factors may have the greatest impact on outcomes, there is broad consensus that tailoring of materials to individual participant characteristics enhances program credibility [52] and is likely to increase program efficacy [53,54].
- The program can monitor which intervention materials each user has accessed in order to encourage participant engagement by presenting fresh, non-redundant content.
- Information from past sessions can be used more efficiently than in a face-to-face setting to reinforce gains made, to shape the subsequent program content, and to provide ongoing feedback [55,56].
- Users can set their own pace and can access information at any time. This “on demand” capability is of particular importance with postpartum women, for whom flexible access to program content is a requirement given their childcare commitments and general lack of time.
- Web forums can provide helpful advice and support within an anonymous environment [35,54].
- There is low post-development cost to deliver the program to each participant.

Guided human support (ranging from a technician-level coach to a more highly skilled therapist) has been shown to increase adherence to online mental health treatments [57,58] and a number of Web-based mental health interventions have used telephones or other mediums to deliver this [20,37,59,60]. Mohr, Cuijpers, and Lehman [57] have developed a robust model (“supportive accountability”) of the mechanisms by which adherence to technology-delivered interventions can be enhanced by human support. Alliance with a “trustworthy” coach is central to this model and a number of studies have now shown that a strong online working alliance can be achieved in guided Internet treatments for PTSD and depression [eg, 48,63]. However, while amount of contact time correlates positively with efficacy [61], there are nevertheless diminishing gains in increasing contact time above a certain threshold [62,63]. Similarly, there is growing evidence that less clinically skilled workers (ie, technicians versus clinicians) can provide sufficient low-intensity support to enhance the therapeutic effects of Internet CBT programs, even in clinically diagnosed samples [60]. The emerging picture is that very encouraging therapeutic effects can be achieved through structured Internet programs, supported by low intensity-guidance (typically <3 contact hours in a six-week program), which has further implications for cost-effectiveness, reach, and widespread dissemination [64].

### Overview of Approach

The *MomMoodBooster* program (USA) and *MumMoodBooster* program (Australia) are Web-based interventions, supplemented with a series of calls with a personal coach, based on an adaptation of Milgrom’s group CBT treatment for postpartum depression. The program was developed by a multinational team composed of researchers based in three organizations: Oregon Research Institute (ORI), Parent-Infant Research Institute (PIRI) in Melbourne, Australia, and the Iowa Depression and Clinical Research Center (IDCRC). This report delineates the formative research procedures used to develop the program content and provides an overview of the eventual design, architecture, and key program ingredients of the *MomMoodBooster* program.

## Methods

As we have described elsewhere [65], the current research is an adaptation of Stage I in the Stage Model of Behavioral Therapies Research [66] and the multistage research model recommended by the USDHHS Science Panel on Interactive Communications and Health [67]. Stage I typically involves formative evaluation to assess the nature of the problem behavior in addition to the needs of the target population in order to inform intervention design and program content. Process evaluation is then used to assess and improve the administrative, organizational, and operational features of the intervention. The current report describes preliminary phases of Stage I research that involved intervention development and formative evaluation that represented a link between feasibility research and subsequent, more controlled research stages. The formative research procedures used to inform the development of the *MomMoodBooster* program involved focus groups and usability testing. We followed an iterative and incremental process in which early feedback was used to accomplish rapid changes [68]. Testing occurred first in Melbourne, Australia, and was followed by testing in Iowa City, Iowa. As a result, the feedback we received in Melbourne was used to further refine the content that was then tested in the subsequent sessions in Iowa.

### Participants

Eligibility criteria for participants in focus group and usability testing were used in order to enroll mothers who shared key characteristics with the participants of our planned feasibility study. Specifically, mothers had to be English-speaking, less than 12 months post partum, at least 18 years of age, have home access to the Internet, and use personal email. They had to report a personal history of depressive episodes in the period following the birth of their baby as indicated by either the Edinburgh Postnatal Depression Scale (EPDS ≥ 12) or the Beck Depression Inventory (BDI-II ≥ 14). The EPDS is a widely used self-rated depression screening assessment that has been validated using various cut-offs [69,70]. Similarly, the BDI is a widely used assessment tool for depression in the postpartum period [71,72]. There were two separate cohorts of participants, corresponding to the design of the subsequent feasibility study that called for participants to be recruited from Iowa and Melbourne, Australia.

Focus groups and usability testing sessions were held in Melbourne, Australia, in late September 2010, followed approximately 2 weeks later by sessions held in Iowa. Eligibility criteria for focus group participants essentially mirrored the eligibility criteria of participants of the eventual feasibility trial of the *MomMoodBooster* intervention. The focus group protocol and related Informed Consent procedure were reviewed and approved by the Human Research Ethics Committee of Austin Health in Australia and the Institutional Review Boards of both ORI and the University of Iowa.

#### Recruitment

Recruitment for the Australian testing used promotion of the study to health professionals and services in the Parent-Infant Research Institute’s (PIRI) existing referral networks, including over 100 Maternal and Child Health Centers (MCHCs) in northern and central metropolitan Melbourne. Women were also recruited from PIRI’s clinic population. Women were screened for eligibility, completed the EPDS, and those meeting eligibility criteria were invited to participate.

Recruitment in Iowa involved sending letters to women listed in the State of Iowa birth registry as having given birth in the last 12 months and who lived within three counties within an hour’s driving distance from the University of Iowa. Research staff then called these women. After consenting to participate, these new mothers were briefly screened for eligibility including completion of the EPDS over the phone. Those mothers who passed the second round of eligibility were then scheduled to participate in a specific focus group. The mothers signed the consent and completed additional questionnaires prior to the start of the focus group. These participants signed the consent and completed additional questionnaires prior to the start of the focus group.

#### Focus Groups

The Australian focus group was conducted at PIRI in Melbourne and involved 8 women who had a mean age of 36.0 years (SD = 5.5 years). All women were Caucasian Australians from mixed ethnic backgrounds as is typical of the population. Childcare resources were available, and participants received $50 for their participation. Each session was audio recorded and transcribed.

The Iowa focus group was held in the Iowa Depression and Clinical Research Center (IDCRC) in Iowa City, IA, and involved 9 women who had a mean age of 29.4 years (SD = 6.29 years). Eight mothers identified themselves as Caucasian, and 1 mother identified herself as African American. Two ORI researchers (BD and JS) participated in all focus group sessions.

#### Usability Testing

The Australian usability testing sessions were conducted at PIRI in Melbourne, Australia, and the Iowa usability testing sessions were held at IDCRC in Iowa City, IA. These sessions typically involved women who had participated in focus groups and were scheduled to occur in the same location and at a time immediately following completion of focus group sessions. This approach ensured that usability testers would have a broad familiarity with the research project and preliminary design ideas about the Web-based intervention. All usability testers met eligibility criteria that mirrored what was planned for participants of the eventual feasibility trial. The mean age of the 14 usability testing participants at PIRI was 36.2 years (SD = 3.9 years), and the mean age of the 8 women at IDCRC was 29.3 years (SD = 6.6 years). Participants received $75 each for their participation.

### Procedures

#### Focus Groups

Our focus group procedures were informed by our experience and by published guidelines [73]. Each session began with participant completion of the Informed Consent followed by a brief pre-assessment. The session lasted approximately 1.5 hours. Focus groups were facilitated by project researchers and observed by additional research team members.

The initial portion of the session provided an overview of the research team followed by a description of the proposed structure and ingredients of the *MomMoodBooster* program including a series of sequential sessions (eg, Getting Started, Managing Your Mood, Increasing your Pleasant Activities), a library of relevant articles, tracking tools, and tools for support. The presentation also highlighted the use of videos, the ability to personalize the webpages with picture files from home, the role of Personal Coach calls, and a possible weekly schedule for sessions that would be flexible to accommodate somewhat longer time periods so that each mother had the opportunity to spend some extra time on any session in order to learn—and use—the recommended strategies.

Mock-ups of selected webpages were presented including a welcome page, a webpage on negative thoughts that prompted discussion about the proposed left navigation features and the contents of the top menu, and a webpage from the tools menu that users would access in order to track daily mood and pleasant activities.

At various points, participants were asked a series of questions designed to elicit opinions and set the occasion for group discussion. For example, following the discussion of the Web forums, participants were asked how often they would post messages to a forum or would choose to read the posts made by other program participants. Following the discussion of the Partner Support program, participants were asked whether they would recommend the Partner Support program to their partner, whether their partner would visit the program, and whether the label “partner” was acceptable for the Partner Support website. Following presentation of the Personal Coach calls, participants were asked whether they thought such calls would be helpful, who should initiate the calls, the preferred characteristics and experience of Personal Coaches, ways calls might be scheduled in order to make them more practical, what to do when scheduled calls were missed, and the length of calls.

At the end of the session, participants were asked to describe their overall reaction to the proposed features of the program (the colors, fonts, imagery, and complexity of the webpages). Candid opinions were encouraged including what participants did not like as well as any features they thought were missing and should be added.

#### Usability Testing

Web-based interventions should embody established usability standards [74,75]. Once functional program components have been created, usability testers can be asked to provide feedback on program completeness and relevance as well as on the extent to which the program functions properly (eg, that buttons work when clicked, that navigation indicators change to properly reflect each participant’s location in the program). Usability testing was scheduled to occur at interim points in the development process to allow incorporation of iterative feedback from usability testers.

A relatively small number of usability testers can provide extremely valuable data that would inform revisions to the program [76]. Each tester met individually with a research staff member who acted as a facilitator. At times, another staff member would be present in an observer role. Usability testers were asked to explore these interactions while receiving minimal direction from the facilitator. Each tester was asked to describe her thoughts using the *think-aloud* technique that was derived from cognitive science [77] and has proven effective in the study of human-computer interactions [78,79]. As noted by Hughes [80], the think-aloud technique provides “…direct, real-time observations of the user rather than self-reports such as surveys” (p. 493). Think-aloud methods assess cognition concurrently with its occurrence. Thus these procedures may be better able to describe the thoughts and attitudes of users [81]. The audio from each usability test session was digitally recorded and transcribed.

We report here upon a preliminary usability test phase in which participants met individually with a facilitator to interact with a series of eight different Web-based interactions planned for the *MomMoodBooster* program that had been selected because they were deemed to be important as well as especially challenging (from a usability perspective). These interactions included mood spirals animation, pleasant activity list, mood *plus* pleasant activity rating form, partner support program, practicing change form on extreme thoughts, daily pleasant activities (pie chart), mood ratings plus pleasant activities line chart, and mood rating form. The usability testing was limited to these key interactions excerpted from the program rather than the program in its entirety.

### Measures

#### Focus Groups

We obtained qualitative data from notes created by research staff who observed the focus group session. Session transcripts were also reviewed by research team members for overarching themes with particular emphasis on suggested changes. All of the focus group transcripts were electronically imported into the qualitative analysis software Atlas.ti (version 6.0) [82]. All participant dialogue was transcribed anonymously. Atlas.ti allows for on-screen coding and aids in managing multiple codes including groups (families) of codes. Atlas.ti also contains an advanced search option (query tool) that facilitates the identification of multiple themes across large amounts of text as well as permitting the analysis of similar participant responses. An open-coding strategy was used based on: (a) the central concepts of the proposed *MomMoodBooster* program, (b) themes that had arisen during the focus groups (suggestions by participants), and (c) codes that emerged during textual analysis. Related codes were then grouped together to create categories and subcategories until all relevant themes had been identified.

#### Usability Testing

Participants were asked to consider all that they had learned about the *MomMoodBooster* program and complete the System Usability Scale (SUS) that we adapted slightly to be appropriate for evaluating the *MomMoodBooster* website (Table 1) [83,84]. Brooke [85] has reported that “…the principal value of the SUS is that it provides a single reference for participants’ views of a product’s usability” (p. 194). The value from a 5-point scale in the 10-item SUS scale describes the features of the website. We followed the recommendation by Brooke [85] and Sauro [86] to convert raw score ratings ranging from 1 to 5 based upon directionality of items.

**Table 1:** System Usability Scale item responses: Combined sample (N= 21).

		Negative			Positive		
		0	1	2		3	4
1	I think that I would like to use this website frequently.			14.3%		47.6%	38.1%
2	I found the website unnecessarily complex.^a^			4.8%		33.3%	61.9%
3	I thought the website was easy to use.			14.3%		28.6%	57.1%
4	I think that I would need the support of a technical person to be able to use this website.^a^					14.3%	85.7%
5	I found the various functions in this website well integrated.		4.8%	19.0%		42.9%	33.3%
6	I thought there was too much inconsistency in this website.^a^			14.3%		28.6%	57.1%
7	I would imagine that most people would learn to use this website very quickly.		4.8%	4.8%		38.1%	52.4%
8	I found the website very cumbersome to use.^a^			9.5%		28.6%	61.9%
9	I felt very confident using the website.	4.8%		4.8%		38.1%	52.4%
10	I need to learn a lot of things before I would get going with this website.^a^					33.3%	66.7%
	Proportion for all participants	0.5%	1.0%	8.6%		33.3%	56.6%

^a^ Values of responses to these items were reversed when scored.

## Results

### Focus Group

**Figure 1:**
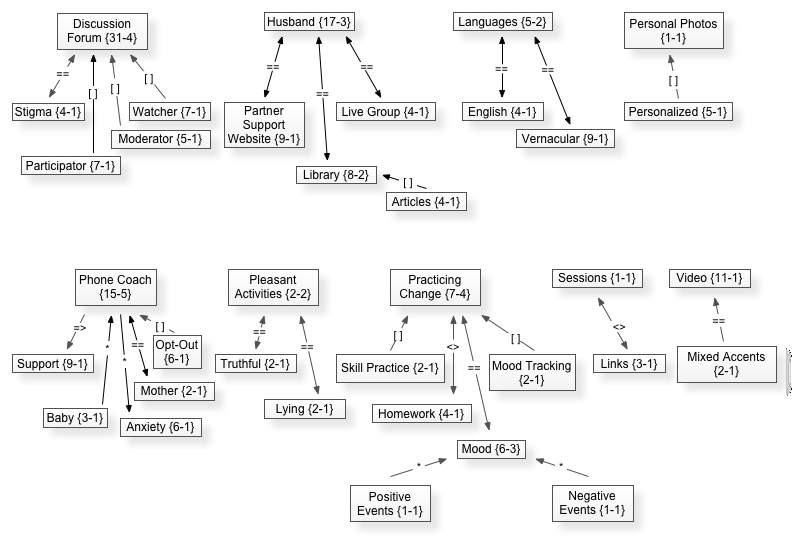
Mock-up of Atlas.ti network view diagram of focus group participant comments showing theme of Engagement.

The network view option in Atlas.ti allowed us to analyze relationships between codes and themes in a visual format. In Atlas.ti, codes are independent of each other until you create a network and “link” the codes together. The links are tagged with unique symbols to delineate what kind of relationship the codes have, ie, affects (*), is a part of ([ ]), is associated with (==), contradicts (<>), etc. This particular network view (Figure 1) is structured around the theme “Engagement” and all of the codes associated to this particular concept.

From this network view diagram (Figure 1), it can be seen that nine themes were discussed relating to engagement. Some examples include participants voicing opinions and concerns related to their husbands’ participation in the program, the expressed benefits and roadblocks they would have with the phone coach aspect of the program, and their suggestions related to the proposed discussion forum. The lines connecting codes in this network view are the relationship links discussed previously. The numbers that appear under (or beside) each code have separate meanings. The first number denotes the number of quotations in the transcripts associated with that code, and the second number denotes how many codes are linked to that code in the network view option of Atlas.ti. For example, the topic of ”Discussion Forum” arose in participants’ discourse 31 times across all of the transcripts, and discussion forum is directly linked to 9 other codes, 3 of which are in this particular network view: stigma, participator, and watcher. The number of times a theme is mentioned in quotations taken from the transcripts provides a possible index of its importance.

#### Participant Comments

Focus group participant comments in both Iowa and Melbourne were quite positive for the most part:

- “I think this is wonderful, because you can do it at home.”
- “Very successful in making me feel confident to use it.”
- “Consider tips for single parents.”
- “The colors and font were very inviting.”
- “The graphics and videos are great, and the anecdotes from other mothers would be brilliant.”
- “It wouldn’t take me long at all to get the hang of it.”
- “I probably wouldn’t click a video.”
- “With this it would be so convenient because it would be right there in my house. I would definitely be a lot more likely to use that.”
- “It’s nice to know that you’re not alone in the universe.”
- Regarding phone coaching: “Having one more thing to do/remember would be difficult...The phone always rings when the baby is screaming.”
- “I really think this is wonderful, because you can do it at home and you don’t have to go somewhere and talk to somebody.”

A list of the overarching themes and summarized participant statements are described in Table 2. In general, mothers indicated support for the web forum and partner support features of the website. The use of multicultural video vignettes was also endorsed by the focus group participants. Participant feedback was also informative with respect to encouraging skill practice (eg, do not refer to practice assignments as “homework”) and the personal coach (eg, assign the same coach for each session; female coach; flexible scheduling).

**Table 2:** Summary of comments from focus group participants.

Theme	Comments
Web Forum	Definitely use
	More likely to read than post
	Like “Ask the Expert” as a reliable source
	Desire moderation/monitoring of posts
Partner Support Program	Most agreed they would invite partner
	Many believe partner would not use support program
	Motivate partner by emphasizing mother wellbeing
	Could facilitate communication between spouses
	The use of the term “partner” was acceptable to most
Videos	Mixture of US & Australia videos would be fine
	Comfort that other cultures experience PPD
	Make program more engaging
Practice Change Activity	Term more acceptable than homework
	Provides encouragement
	Need flexible schedule
	Keep simple
	Reminder would be helpful; on website or email
Personal Coach	Prefer same coach each call (all participants agree)
	Woman-Mom would be preferable as coach
	Coach should call participant
	Flexible scheduling
	Use email reminders
	Pick a set duration; anything under a half hour OK
	Could opt out without being dropped from program
	Strongly suggest call 2 times before discontinuing
Tools	Generally like the idea of daily tracking
General	Name and colors agreeable

### Usability Testing

#### Quantitative Results

SUS ratings (0 = extremely negative; 4 = extremely positive) were obtained for 21 of 22 (95.5%) of the usability testers: the Melbourne sample (N = 14; mean = 3.36; SD = 0.38) and the Iowa sample (N = 7; mean= 3.66; SD = 0.36). The combined samples had a SUS score mean of 3.45 (SD = 0.39). It is helpful to examine the proportion of responses across participants by the assigned adjusted SUS ratings (see Table 1). Using this perspective, we note that 33.3% of testers assigned a score of 3, and 56.6% of respondents assigned a score of 4 that resulted in 90.9% of respondents having a positive reaction to the usability of the *MomMoodBooster* program.

**Figure 2:**
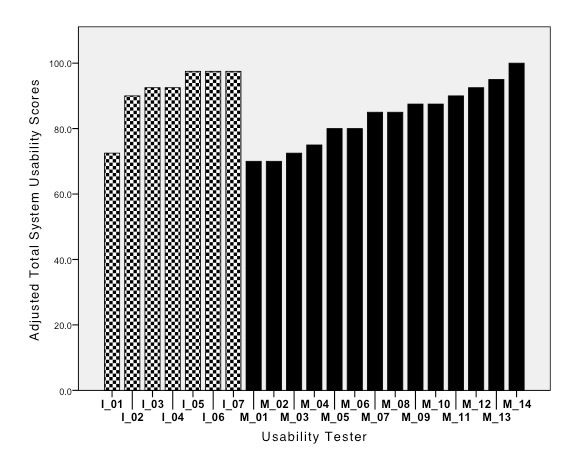
Overall adjusted System Usability Scale scores (N= 21) (patterned fill= Iowa usability testers; solid fill= Melbourne usability testers).

Results for the SUS total scores that had a range of 0 to 100 were as follows: for the Melbourne sample (N = 14; mean = 83.57; SD = 9.39), for the Iowa sample (N = 7; mean = 91.43; SD = 8.88), and for the combined samples (N = 21; mean = 86.19; SD = 9.77). As shown in Figure 2, the individual SUS are quite uniform with scores greater than 80 being reported by 14 of the 21 participants (67%).

A SUS score of 73 has been described as good, a score of 85 as excellent, and a score of 100 as the best imaginable [86]. Using the scoring interpretation for the overall adjusted SUS score results obtained, it appears that each of the usability testers found the selected interactions from the *MomMoodBooster* program to be very usable.

#### Qualitative Results

As part of *thinking aloud*, usability testers at times shared evaluative comments about program features. Examples included the following:

- “I love that I can click on this and make it big, so I can see it and understand it. Ooh, the pie chart cracks open. I like this, this is really cool.”
- “Red text draws attention; I don’t need to know what I did not do. Get rid of red.”
- “It’s very good. It’s [a] very easy program, it doesn’t have a lot of—you know how sometimes you get on web pages and there’s so much to see, and it seems like every time you go to the spot you need to go to, you always need to go back four pages. It doesn’t seem to have any of that sort of stuff.”
- “I do like the partner part…I think that coming to see the psychologist, I think my partner missed out, and he would have liked to have known—he was so scared of what was being said.”
- “A lot of reading here. He would not read this much.”
- “It was good, actually, because it sounds like it’s a really useful tool for mothers.”
- “Chunks of writing [are] too much; needs colors to stand out more.”

Feedback in terms of tester comments and the observations of the research staff who facilitated the formative sessions led to myriad important refinements to the eventual design of the *MomMoodBooster* program. For example, we added language and tone gleaned from comments shared in focus group and usability testing sessions to enhance the relevance of our content and increase the credibility of our program. Portions of some of the stories shared in the focus group were used as the basis of stories presented in our online video vignettes. The eventual protocol we used for coordinating calls between personal coaches and program participants took into consideration the opinions mothers shared with us in focus group sessions regarding the fact that the Personal Coaches should initiate the calls, but mothers have an online method to share scheduling messages with their Coach.

Usability feedback about challenges with our initial online tracking tools led us to further simplify their interface and add more context-sensitive user instructions. Based on comments we received, the Practice Change (homework) activities in the program became key components that encouraged home practice of the recommended behavioral skills. Because participants emphasized the importance that their Practice Change activities along with their other progress in the program should be available to Personal Coaches to enhance the relevance of their shared calls, a sophisticated administration website was created that provided Personal Coaches with a digital dashboard showing details of participant progress in using the *MomMoodBooster* program.

### MomMoodBooster Program Design

**Figure 3:**
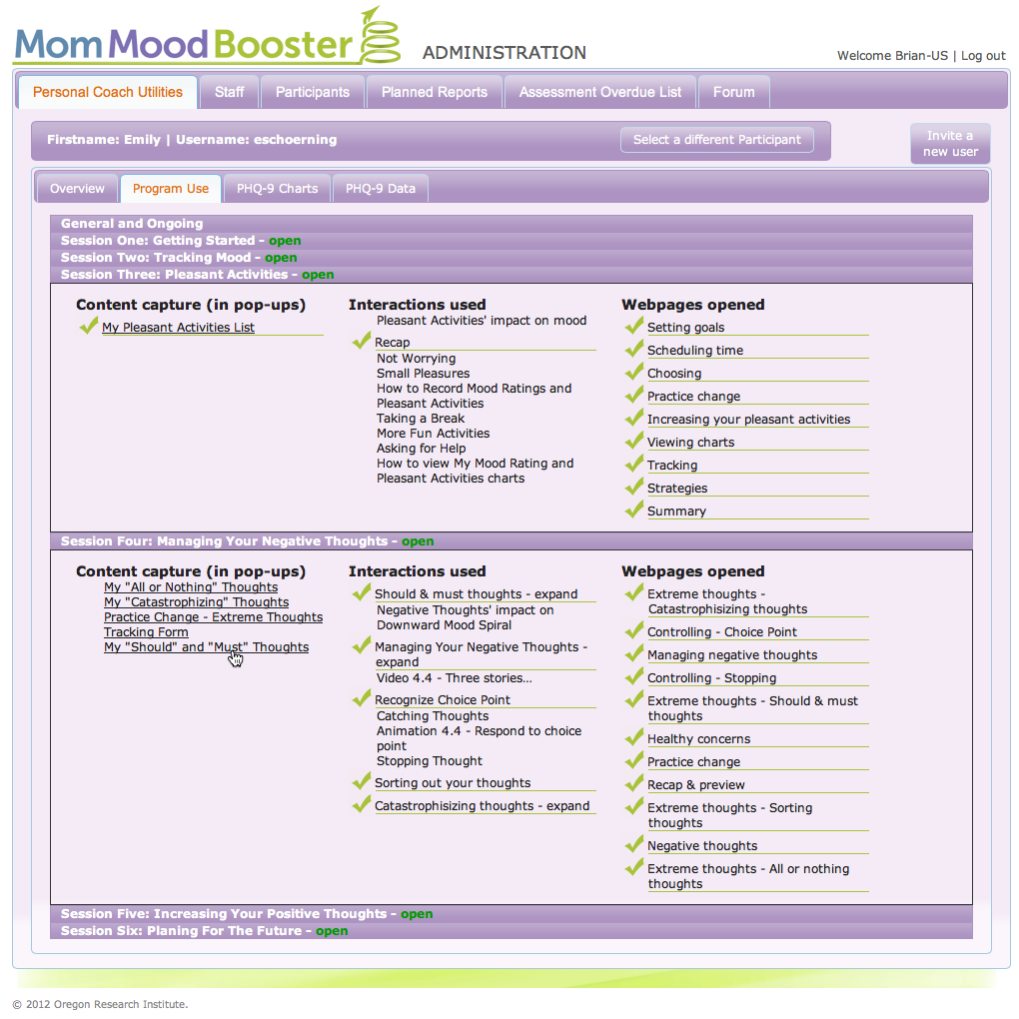
Dashboard for Personal Coach Webpage in MomMoodBooster Administrative Website (checkmarks indicate that participant has used certain interactions or viewed specific webpages).

**Figure 4:**
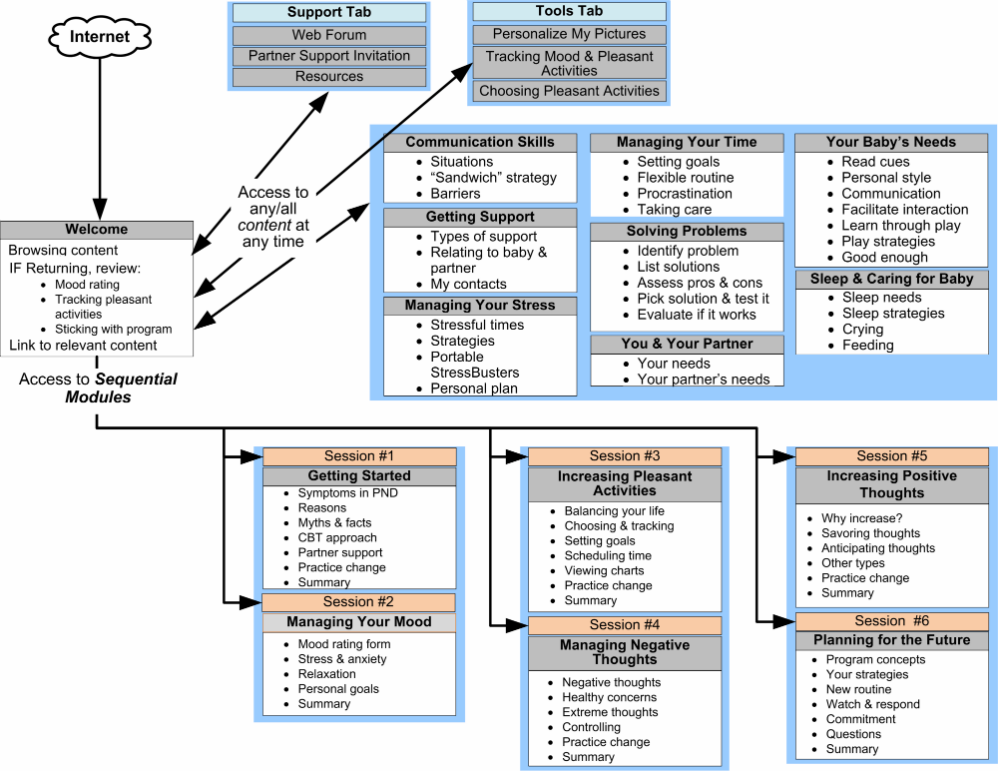
Structure of MomMoodBooster program.

**Figure 5:**
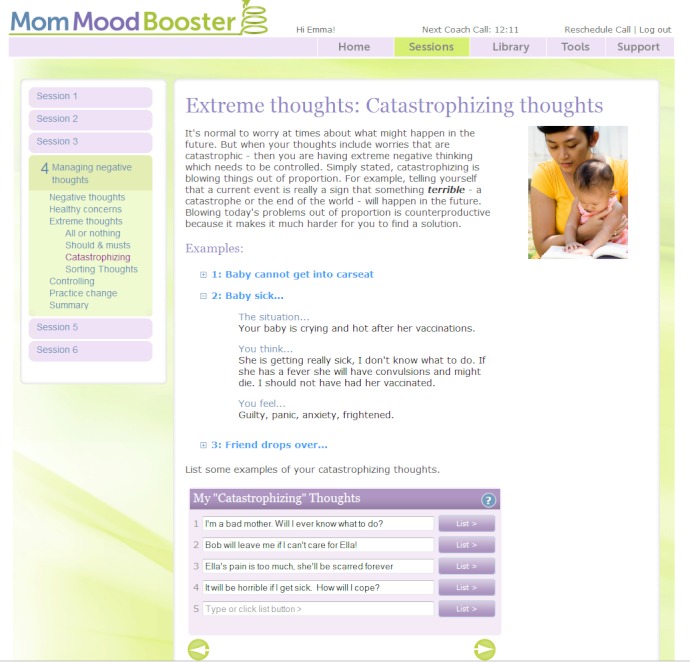
Webpage from MomMoodBooster Session 3 (Displays content participant has entered for personal list of Catastrophisizing Thoughts).

The resultant *MomMoodBooster* program combines a tailored, interactive Web-based postpartum depression intervention for individual mothers who also receive a series of Personal Coach calls. The overall program also includes two additional websites: a Partner Support website and an Administrative website that includes a dashboard for Personal Coaches (Figure 3). A schematic depiction of the final content design of the *MomMoodBooster* program is shown in Figure 4. The program guides participants through a series of six sequential sessions: (1) Getting Started, (2) Managing Mood, (3) Increasing Pleasant Activities, (4) Managing Negative Thoughts (example webpage shown in Figure 5), (5) Increasing Positive Thoughts, and (6) Planning for the Future. The program follows a schedule that makes available each successive session across a 6-week period. Each session opens with an auto-play video introduction of session goals and content provided by the program host. Webpages in each Session use text, interactions, animations, and video to present program content.

**Figure 6:**
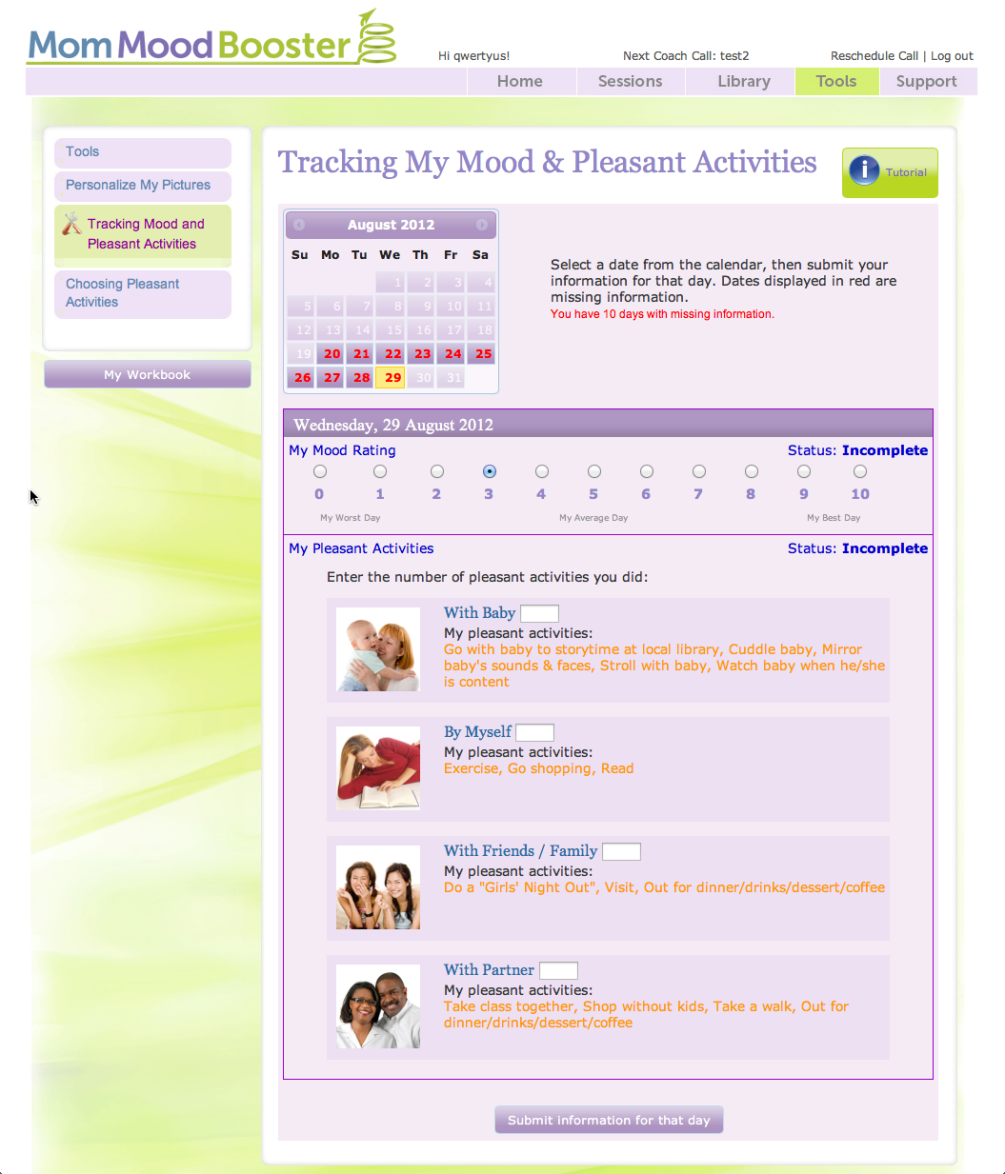
MomMoodBooster Tracking Tools used daily to track Mood Ratings and number of personally-selected Pleasant Activities accomplished.

The secure program encourages participants to personalize their program content by typing in their personal lists, setting personal goals, and performing *practice change* activities in their everyday routines. Mothers can print out a personal workbook that summarizes their personalized content while also providing a brief written record of the content they covered. *MomMoodBooster* program users can further personalize their program by uploading their own photos so that they are displayed on program webpages that they view. The program includes self-monitoring tools that enable the daily tracking and online charting of both mood and pleasant activities (Figure 6) and other online resources. It also includes ad hoc (anytime) access to a library of relevant articles on communication skills, getting support, managing stress, managing time, solving problems, sleep and caring for baby, baby’s needs, and your partner.

Since social isolation and stigma are often experienced by depressed mothers with newborn babies, the *MomMoodBooster* program provides access to a private peer-based Web forum in which mothers can post a message as well as read and interact with the messages of other participants.

The *MomMoodBooster* program includes a weekly phone call from an assigned Personal Coach. This role is designed to be largely non-therapeutic as it is intended only to provide support, encourage program engagement, and clarify how best to use the online program. Personal Coaches can access a special digital dashboard that describes the extent that an assigned participant has interacted with the program (Figure 3). During bi-weekly coach calls study participants are asked to complete the PHQ-9 assessment, which is used as a safety check of each participant’s status [87]. Deterioration in PHQ-9 scores of ≥ 20% of baseline is used to trigger a participant safety procedure that was well-established in both research sites (Melbourne and Iowa).

Finally, because partners/fathers also have an important role to support mothers in working with the program, the program provides an email feature to enable mothers to invite their partners to visit a separate Web-based partner support program that describes postpartum depression, the *MomMoodBooster* program, and ways they can be supportive.

## Discussion

Postpartum depression represents an important public health problem especially because many new mothers are unable or reluctant to seek help. Poor treatment uptake results in many mothers not accessing services or receiving support to ameliorate their depression. This can have substantial consequences for themselves, their partners, and their infants. Web-based depression interventions represent a rapidly emerging approach for extending the reach of efficacious treatments [64,88]. The *MomMoodBooster* program is a highly innovative Web-based intervention specifically designed to ameliorate postpartum depression. In this report, we have described: (1) the formative work that contributed to the development of our Web-based intervention for helping to relieve symptoms of postpartum depression, and (2) the design and key components of the program.

The qualitative and quantitative data gathered in the focus groups and usability sessions reported here suggest that the new mothers involved had largely positive reactions to the major features of the *MomMoodBooster* program and that those program features performed well in terms of usability. Specifically, the formative results supported our use of sequential sessions, involvement of the Personal Coach having certain characteristics, the availability of a separate support website for the partner, etc. In addition, we found that using the ATLAS.ti qualitative analysis tool helped to identify useful themes from our focus group session transcripts.

Limitations of the current formative research are largely related to the fact that our focus group and usability testing samples were not representative of all mothers nor did all participants provide us with a complete data set. Although participants in the formative research satisfied eligibility criteria similar to those we intended to use for participants in our planned feasibility study, this group of women is best considered a convenience sample that may not generalize to all postpartum mothers. For example, the Australian sample was somewhat older (focus group: M = 36 years, SD = 5.5) than the median age of mothers in Australia (2010 births; 30.7 years) but more consistent with Australian women ages 30-34 years who have the highest fertility rate of all age groups in that country [89]. The Australian sample was also older than the American participants, which is consistent with 2010 birth data for both countries (in Australia, 51% of mothers giving birth were 30-39 years old [89], whereas in the US that age range accounted for only 36% of mothers giving birth [90]). We did not collect data on participant education or income, which prevents any discussion of representativeness of our sample.

It is also important to acknowledge that the usability test results described in this report were obtained in a preliminary usability phase. Our usability testing efforts did not end at this initial step in the process. Instead, usability feedback continued to occur in an iterative and incremental manner as the *MomMoodBooster* program continued to be developed [68]. In addition, participants who provided SUS ratings at this initial phase had only relatively brief interactions with preliminary designs of key program tools and interactions. We anticipate that positive SUS results will also be obtained from feasibility study participants who use the completed, fully integrated *MomMoodBooster* program.

In terms of lessons learned, we concluded that there were many more similarities than differences between our Australian and American samples of postpartum mothers. There were differences in language in terms of spelling and in terms of common usage. We concluded that these differences were critically important features that need to be localized in the final Web-based program. But some differences in language and accent did not preclude the use of Australian videos and audios with an American audience and vice versa. As more than one participant indicated, they felt less isolated knowing that mothers from the other cultures shared similar challenges with postpartum depression. Finally, the technological sophistication of our two participant samples was quite equivalent. These similarities provide support for expanded US/Australia collaborative research on Web-based interventions.

The *MomMoodBooster* program is currently being tested in a feasibility trial that involves 50 depressed postpartum mothers—25 in Australia using the *MumMoodBooster* program and 25 additional mothers in Iowa using the *MomMoodBooster* program. While the content of the two programs is identical in meaning, each version uses country-specific language, and each has its own set of host videos for every session. The two programs use Personal Coaches and they also share video vignettes of mothers describing their implementation of some of the strategies using a mixture of American and Australian actors. It remains for the results of our feasibility trial and a subsequent fully powered RCT to establish the efficacy/effectiveness of the *MomMoodBooster* program. Results from our formative research have been highly encouraging and were instrumental in developing a program acceptable to mothers with PPD.

## Abbreviations

BDI: Beck Depression Inventory
CBT: Cognitive Behavioral Therapy
CWD: Coping with Depression course
EPDS: Edinburgh Postnatal Depression Scale
IDCRC: Iowa Depression and Clinical Research Center
ORI: Oregon Research Institute
PHQ-9: Patient Health Questionnaire (9-item)
PIRI: Parent-Infant Research Institute
PPD: Postpartum Depression
PTSD: Post-Traumatic Stress Disorder
RCT: Randomized Controlled Trial
SUS: System Usability Scale
